# Prolonged Elevation of Arterial Stiffness Following Peak Aerobic Exercise in Individuals With Chronic Stroke

**DOI:** 10.3389/fphys.2021.666171

**Published:** 2021-05-17

**Authors:** Kenneth S. Noguchi, Kevin Moncion, Elise Wiley, Maureen J. MacDonald, Julie Richardson, Marc Roig, Ada Tang

**Affiliations:** ^1^School of Rehabilitation Science, McMaster University, Hamilton, ON, Canada; ^2^Department of Kinesiology, McMaster University, Hamilton, ON, Canada; ^3^Memory and Motor Rehabilitation Laboratory, Feil and Oberfeld Research Centre, Jewish Rehabilitation Hospital, Montreal Center for Interdisciplinary Research in Rehabilitation, Laval, QC, Canada; ^4^School of Physical and Occupational Therapy, McGill University, Montreal, QC, Canada

**Keywords:** arterial stiffness, stroke, aerobic exercise, hemodynamics, heart rate, blood pressure

## Abstract

**Background:**

Stroke is a highly disabling condition and is the second leading cause of death globally. Engaging in aerobic exercise is important for the prevention of a recurrent stroke through improving markers of cardiovascular health such as blood pressure and arterial stiffness. While higher intensities of aerobic exercise generally elicit greater cardioprotective effects, little is known about the acute cardiovascular effects of a single session of high intensity aerobic exercise in people with stroke. The objective of this study was to model the recovery of arterial stiffness (carotid-femoral pulse wave velocity, cfPWV), heart rate and blood pressure following peak intensity aerobic exercise in individuals with chronic stroke.

**Methods:**

Ten participants with chronic stroke (mean ± SD age = 56.9 ± 11.8 years, median [IQR] years post-stroke = 2.9 [1.9]) performed a symptom-limited cardiopulmonary exercise test (CPET) on a recumbent stepper. Before the CPET, resting cfPWV, heart rate and blood pressure were measured. Immediately following the CPET, all outcomes were measured again continuously for 20 min to use all available observations (*n* = 245 observations) and capture any potential non-linear changes. Mixed model analyses were then applied to model post-exercise changes of cfPWV, heart rate and blood pressure.

**Results:**

Carotid-femoral pulse wave velocity was increased from rest following the CPET (9.0 ± 0.53 to 9.9 ± 0.52 m/s, *p* < 0.001) and remained elevated for 20 min into post-exercise recovery, independent of heart rate (*p* = 0.001). Heart rate also increased from baseline (71.2 ± 3.2 to 77.4 ± 3.1 bpm, *p* < 0.001) and remained elevated for 10 min post-exercise (*p* < 0.001). Finger systolic blood pressure was reduced from rest (117.3 ± 4.7 to 111.8 ± 4.6 mmHg, *p* < 0.001) and remained reduced for 15 min after exercise (*p* < 0.001). There were no significant differences in finger diastolic or mean arterial pressures from rest.

**Conclusion:**

This was the first study to capture continuous changes in cfPWV following peak aerobic exercise in any clinical population. The present study revealed that cfPWV is elevated for 20 min after peak aerobic exercise in individuals with stroke, which was independent of heart rate. These findings suggest there may be autonomic imbalances in large arteries following peak intensity aerobic exercise in individuals with stroke.

## Introduction

Stroke is the second leading cause of death globally and affects over 80 million individuals worldwide (Johnson et al., 2019). Risk factors for primary stroke are well established and include current smoking, diabetes mellitus and hypertension (O’Donnell et al., 2010). Novel risk markers have also been identified to predict future stroke risk. One such marker is arterial stiffness, which is the product of complex interactions of structural and cellular mechanisms in the arterial wall (Zieman et al., 2005). Arterial stiffness has since emerged as a strong independent risk marker for stroke (van Sloten et al., 2015) and is a predictor of poor functional recovery post-stroke (Gąsecki et al., 2012).

Aerobic exercise is an established as an effective method for managing traditional risk factors for index and recurrent stroke (Lin et al., 2015; D’Isabella et al., 2017), but the effects of aerobic exercise on novel markers like arterial stiffness are less well established in the stroke population. Carotid-femoral pulse wave velocity (cfPWV), the criterion standard assessment of arterial stiffness, is negatively associated with cardiorespiratory fitness in individuals with stroke (Tang et al., 2014); thus, it is likely that aerobic exercise training also promotes novel markers cardiovascular health.

It is possible that acute perturbations can have opposing effects on cfPWV. Historically, acute increases in cfPWV were thought to be caused exclusively by increases in aortic blood pressure (Chirinos, 2012). However, it has recently been shown that increases in cfPWV are likely to be caused by sympathetic nervous system activation after an acute stimulus and can occur independently of blood pressure (Faconti et al., 2019). Thus, it is unsurprising that acute bouts of high-intensity aerobic exercise can cause transient increases in markers of arterial stiffness. The acute increases in cfPWV typically returns toward, and even below, baseline levels after 5 min of post-exercise recovery in the general population (Mutter et al., 2017). However, among adults with impaired cardiovascular profiles such as hypertension (Gkaliagkousi et al., 2014), obesity (Bunsawat et al., 2017), and smokers (Doonan et al., 2011), elevations in cfPWV after peak-intensity aerobic exercise persist for longer than 10 min. Prolonged exposure to elevated cfPWV following acute exercise has been associated with increased cerebrovascular pulsatility, which represents hypoperfusion to the cerebrovasculature (Kassab et al., 2007), and thus increases risk for adverse cerebrovascular health (Lefferts et al., 2018). Overall, it is important to understand acute exercise-induced changes to large artery stiffness in individuals with stroke, and for periods longer than 10 min post-exercise.

The objective of this study was to model the recovery of cfPWV, heart rate and blood pressure following peak intensity aerobic exercise in individuals with chronic stroke. Based on previous evidence in adults with risk factors for cardiovascular disease (Doonan et al., 2011; Gkaliagkousi et al., 2014; Bunsawat et al., 2017), we hypothesized that cfPWV would remain elevated for ≥10 min beyond cessation of peak exercise, independent of heart rate in individuals with stroke.

## Materials and Methods

### Study Design and Setting

This study was a cross-sectional analysis using baseline data from two clinical trials: a randomized controlled trial comparing the effects of high-intensity interval training to moderate intensity continuous training on cardiovascular and functional outcomes after stroke, and a prospective, single group study comparing the cardiovascular response to acute peak exercise, high intensity interval exercise and moderate intensity continuous exercise. Both studies were approved by the local university institution research ethics board (Hamilton Integrated Research Ethics Board #3113, #4713) and registered in a clinical trials registry (NCT03570216, NCT03614585). All participants provided informed, written consent.

### Participants

Participants for both studies were recruited from local community stroke recovery groups and from a database of participants who previously participated in studies from the lab and consented to be contacted for future research.

Eligibility criteria for both studies were similar. Individuals were invited to participate if they were (1) 40 to 80 years old, (2) ≥6 months following first-ever, single stroke confirmed by MRI or CT scan, (3) living in the community, and (4) able to walk ≥ 10 m with or without the use of a gait aid but without the assistance of another person. Individuals were excluded if they (1) experienced a tumor or stroke of non-cardiogenic origin, (2) had moderate to severe disability following stroke, defined by a modified Rankin scale score > 2, (3) were actively engaged in stroke rehabilitation services, (4) were class C or D American Heart Association Risk Score, (5) had any neurological or musculoskeletal condition precluding safe exercise, (6) experienced pain that worsened with exercise, or (7) had any contraindications to exercise testing or training as outlined by the American College for Sports Medicine (Riebe et al., 2018).

### Experimental Design

Participants arrived to the lab fasted for ≥4 h, did not exercise for ≥24 h prior to the study visit, and were instructed to take their medications as prescribed to limit the risk of adverse events. Participant demographics including age, biological sex, details of stroke such as type, location and date of stroke, and other relevant medical history were recorded at the beginning of the visit. Participants were assessed for stroke severity using the National Institutes of Health Stroke Scale (Brott et al., 1989; maximum score: 42) and the modified Rankin Scale (Van Swieten et al., 1988; maximum score: 6); higher scores on both assessments indicate greater severity.

Two resting supine measurements of cfPWV, heart rate and blood pressure were then collected after ≥10 min of rest in a temperature-controlled room. The mean of the two measurements were taken to represent baseline levels. Approximately 5 min after the acute exercise stimulus, cfPWV, heart rate and blood pressure were continuously collected until 20 min into exercise recovery.

### Acute Exercise Stimulus

A symptom-limited cardiopulmonary exercise test (CPET) was used as the exercise stimulus from which we examined the exercise response and recovery of cfPWV, heart rate and blood pressure. The CPET was performed on a recumbent stepper (NuStep T4r, NuStep LLC, Ann Arbor, MI, United States), using an incremental protocol validated for individuals with stroke, beginning with a 4-min warm-up at approximately 20 watts, followed by incremental increases in resistance every 2 min for a maximum of 7 stages at a minimum cadence of 80 steps per minute (Billinger et al., 2008). This specific CPET protocol has been used by many (Nepveu et al., 2017; Wilson et al., 2017; Johnson et al., 2020) to accommodate a wide range of motor abilities in individuals with stroke. Peak oxygen consumption (VO_2_peak, mL/kg/min) was also collected using a metabolic mixing chamber system (model Quark CPET, COSMED SrI, Rome, Italy). Continuous heart rate data was collected (Polar H10 Heart Rate Sensor, Polar Electro, Kempele, Finland) throughout the test. Blood pressure (Dinamap V100, General Electric Healthcare, Chicago, IL, United States) and ratings of perceived exertion scale (Borg, 1982) were monitored at the end of each stage.

The tests were terminated if participants reached volitional fatigue, were unable to consistently sustain the appropriate cadence and/or achieved any of the American College of Sports Medicine test termination criteria for exercise testing (Riebe et al., 2018). Immediately after the CPET, participants rested for 1 min. A cool-down at a self-selected cadence was permitted for up to 5 min to accommodate variability in participants’ fitness levels and lower-limb function.

### Cardiovascular Assessments

#### Central Arterial Stiffness

Central arterial stiffness was determined using the criterion standard cfPWV, following procedures outlined in published recommended guidelines (Wilkinson et al., 2010). Both pre- and post-exercise cfPWV was measured via applanation tonometry (SPT-301, Millar Instruments Inc., Houston, TX, United States) on the carotid and femoral arteries on the participant’s non-paretic side in a supine position. Post-exercise assessment of cfPWV has previously shown to be reliable in older adults (ICC = 0.84–0.94; Perissiou et al., 2019). In accordance with the ARTERY Society guidelines (Wilkinson et al., 2010), a minimum of 10 consecutive and consistent pulse pressure waveforms were collected simultaneously by two experienced testers (KN and KM). The sampling rate was 2 kHz, and band-pass filtered at 5–30 Hz to identify the foot of each waveform (LabChart7 Pro, ADInstruments, Colorado Springs, CO, United States). cfPWV was then calculated as the time delay between the foot of the carotid and the femoral waveforms, averaged across at least 10 consecutive cardiac cycles and subsequently divided by 80% of the measured distance between both sampling sites.

#### Heart Rate and Blood Pressure

Resting and post-exercise values for heart rate, systolic blood pressure, diastolic blood pressure and mean arterial pressure were continuously measured using a three-lead electrocardiograph system (Dual Bio Amp FE232, ADInstruments) and a beat-to-beat finger blood pressure monitoring system (Finometer MIDI, Finapres Medical Systems, Amsterdam, Netherlands).

### Statistical Analyses

Descriptive statistics using means and standard deviations, or medians and interquartile ranges were used to describe sample characteristics for continuous data with normal and non-normal distribution, respectively. Frequencies and percentages were used to describe categorical data.

Mixed-model analyses were used to characterize the response of cfPWV, heart rate and blood pressure following peak aerobic exercise in individuals post-stroke. First, lowess plots were visually inspected to assess the relationship between the dependent (cfPWV, heart rate, systolic blood pressure, diastolic blood pressure, and mean arterial pressure) and independent (time post-exercise, in minutes) variables. If the relationship appeared to be non-linear, polynomial terms were considered in the analysis. If polynomial terms were added to the models, lower-order independent variables will remain in the model in accordance with the hierarchy principle of modeling, irrespective of statistical significance. Age was included as a covariate in all models due to its known relationship with arterial stiffness (Engelen et al., 2015). Heart rate was included as a covariate in the cfPWV model as it is known to influence changes in cfPWV, independently of blood pressure (Tan et al., 2016). Models were tested for both fixed and random intercepts and slopes, and the most appropriate residual covariance structures were applied. Level 1 and 2 residuals were examined for potential outlier observations and were removed from analysis if they had a substantial (>10%) influence on the beta-coefficient. The Bayesian Information Criterion and log likelihood ratio tests were used to determine the best fitting mixed model. If the model was statistically significant, pairwise comparisons using a Sidak correction and effect size estimates were used to determine when differences from rest were present after exercise (Kowalchuk and Keselman, 2001). The accepted two-tailed significance level was set *a priori* to *p* < 0.05. All statistical analyses were performed using Stata 16.1 (College Station, TX, United States).

## Results

### Participant Eligibility

Of 44 individuals examined for eligibility (randomized controlled trial *n* = 33, prospective single group study *n* = 11), 10 were included in the study [reasons for ineligibility: did not meet criteria for age (*n* = 7), history of TIA and not stroke (*n* = 5), more than one stroke (*n* = 3), length of time post-stroke (*n* = 13), walking ability (*n* = 1), or American Heart Association Risk Score classification (*n* = 3), or presented with severe cognitive impairment (*n* = 2)].

### Participant Characteristics

Participants’ characteristics are presented in Table 1, for the entire cohort and disaggregated by sex. There were no differences between males and females across all variables. Although the National Institutes of Health Stroke Scale suggests mild stroke severity, VO_2_peak from the symptom-limited CPET was 59.8 ± 0.2% and 78.2 ± 0.2% of normative values for males and females, respectively, (Riebe et al., 2018) and 6-min walk test distances were 66.4 ± 10.0 and 100 ± 6% of normative values for males and females, respectively, (Enright and Sherrill, 1998). From the CPET, peak values for brachial systolic blood pressure was 183.8 ± 26.6 mmHg, heart rate was 135.9 ± 26.9 bpm (81% of age-predicted maximal heart rate), and respiratory exchange ratio was 0.95 ± 0.08. There were no adverse effects of the CPETs.

**TABLE 1:** Participant characteristics for entire sample (*n* = 10) and disaggregated by sex.

Variable	Total *n* = 10	Females *n* = 4	Males *n* = 6
Age (years)	56.9 ± 11.8	55.5 ± 13.3	57.8 ± 11.9
Type of stroke, *n* (%)			
Ischemic	6 (60%)	2 (50%)	4 (67%)
Haemorrhagic	0 (0%)	0 (0%)	0 (0%)
Unknown	4 (40%)	2 (50%)	2 (33%)
Time post-stroke (years), median (IQR)	2.9 (1.9)	2.8 (7.9)	3.1 (2.3)
Comorbidities, *n* (%)			
Hypertension	7 (70%)	2 (50%)	5 (83%)
Type 2 diabetes	3 (30%)	0 (0%)	3 (50%)
Medications, *n*(%)			
β-blocker	1 (10%)	0 (0%)	1 (17%)
ACE-inhibitor	4 (40%)	0 (0%)	4 (67%)
Calcium-channel blocker	4 (40%)	1 (25%)	3 (50%)
ASA	4 (40%)	2 (50%)	2 (33%)
Body mass index (kg/m^2^), median (IQR)	28.2 (4.6)	27.9 (3.2)	29.2 (4.8)
Resting heart rate (bpm)	69.9 ± 10.6	72.2 ± 8.1	68.3 ± 12.5
Resting systolic blood pressure (mmHg)	125.8 ± 12.6	126.8 ± 15.1	125.2 ± 12.2
Resting diastolic blood pressure (mmHg)	73.3 ± 5.6	70.0 ± 2.2	75.4 ± 6.3
Peak heart rate (bpm)	135.9 ± 26.9	153.8 ± 23.0	124.0 ± 23.7
Peak systolic blood pressure (mmHg)	183.8 ± 26.6	186.2 ± 22.3	182 ± 31.2
Peak diastolic blood pressure (mmHg)	84.5 ± 7.4	86.5 ± 6.7	83.2 ± 8.1
VO_2_peak (mL/kg/min)	19.0 ± 5.5	18.3 ± 5.2	19.5 ± 6.2
Peak respiratory exchange ratio	0.95 ± 0.08	0.99 ± 0.12	0.92 ± 0.05
Resting carotid-femoral pulse wave velocity (m/s)	9.0 ± 2.1	9.6 ± 2.4	8.6 ± 1.9
Time post-exercise of 1st CV measure (min)	5.9 ± 1.7	4.7 ± 0.4	6.5 ± 1.8

*Values are mean ± SD unless otherwise stated. SD, standard deviation and IQR, interquartile range.*

### Changes in cfPWV, Heart Rate and Blood Pressure

On average, the first measures of cfPWV, heart rate and blood pressure were attained at 5.9 ± 1.7 min after termination of the CPET (Table 1). Cardiovascular measures were then captured continuously for 15 min thereafter, and 241–256 observations were obtained for each variable across 10 participants (i.e., 24–26 observations per participant, per variable).

Upon visual inspection of lowess plots, the relationship between cfPWV and time post-exercise appeared to be non-linear. Thus, time^2^ and time^3^ terms were included in the model to capture the polynomial shape. Results of the mixed model are presented in Table 2, where the best fitting model included age and heart rate as covariates, and a random slope and intercept. There were no statistical outliers in this model.

**TABLE 2:** Mixed model analysis describing the changes in carotid-femoral pulse wave velocity after acute, high-intensity aerobic exercise (*n* = 10, 256 observations).

*Fixed effects variables*	β(*SE*)	*95% CI*	*p*
Time post-exercise	0.26 (0.06)	0.14, 0.37	<0.001**^‡^**
Time post-exercise^2^	–0.02 (0.006)	–0.03, –0.01	<0.001**^‡^**
Time post-exercise^3^	0.0005 (0.0001)	0.0002, 0.009	0.001**^†^**
Covariate: Age	0.14 (0.04)	0.05, 0.23	0.004**^†^**
Covariate: Heart rate	0.02 (0.007)	0.007, 0.03	0.27
Constant	0.55 (2.83)	–5.01, 6.11	0.85

***Random effects parameters***	***Estimate*(*SE*)**	***95% CI***	

Slope	0.001 (0.0007)	0.0002, 0.004	
Intercept	2.61 (1.22)	1.05, 6.52	
Residual	0.25 (0.02)	0.21, 0.30	

***Model fit statistics***	***Statistic***		

Log likelihood	−219.5		
Bayesian information criteria	488.9		
Pseudo-*R*^2^	0.11		

CI, Confidence interval and SE, Standard error, significant at *p < 0.05, **^†^***p* < 0.01, and **^‡^***p* < 0.001.

With the exception of diastolic blood pressure, all relationships (heart rate, systolic blood pressure, and mean arterial pressure) with time post-exercise were also non-linear. Thus, polynomial terms (time^2^, time^3^) were included in these models (Table 3). The best fitting age-adjusted model for heart rate included a random slope and intercept, whereas models for systolic blood pressure, diastolic blood pressure and mean arterial pressure each only included a random intercept. We tested unstructured, exponential and independent covariance structures for each model. The best fitting model for cfPWV and hemodynamic variables utilized independent covariance structures. There was 1 statistically outlying observation for the heart rate model, 4 in the systolic blood pressure model, none in the diastolic blood pressure model, and 2 in the mean arterial pressure model (Table 3).

**TABLE 3:** Mixed model analyses on changes in central hemodynamics after peak aerobic exercise.

(A) Heart rate (*n* = 10, 255 observations)			

*Fixed effects variables*	β(*SE*)	*95% CI*	*p*
Time post-exercise	2.17 (0.20)	1.77, 2.57	<0.001**^‡^**
Time post-exercise^2^	−0.21 (0.02)	−0.25, −0.17	<0.001**^‡^**
Time post-exercise^3^	0.005 (0.006)	0.004, 0.006	<0.001**^‡^**
Covariate: Age	−0.003 (0.29)	−0.58, 0.51	0.91
Constant	73.0 (16.1)	41.4, 104.7	<0.001**^‡^**
***Random effects parameters***			
Slope	0.03 (0.02)	0.01, 0.09	
Intercept	95.8 (43.4)	39.4, 233.0	
Residual	3.96 (0.37)	3.30, 4.75	

***Model fit statistics***	***Statistic***		

Log likelihood	−576.9		
Bayesian information criteria	1198.1		
Pseudo-*R*^2^	0.16		

**(B) Systolic blood pressure (*n* = 10, 241 observations)**			

***Fixed effects variables***			
Time post-exercise	−1.42 (0.23)	−1.87, −0.97	<0.001**^‡^**
Time post-exercise^2^	0.06 (0.01)	0.05, 0.08	<0.001**^‡^**
Covariate: Age	0.73 (0.41)	−0.07, 1.52	0.07
Constant	76.6 (23.6)	30.3, 122.8	0.001**^†^**
***Random effects parameters***			
Intercept	204.7 (93.1)	83.9, 499.0	
Residual	23.8 (2.21)	19.8, 28.6	

***Model fit statistics***	***Statistic***		

Log likelihood	−749.3		
Bayesian information criteria	1531.5		
Pseudo *R*^2^	0.12		

**(C) Diastolic blood pressure (*n* = 10, 245 observations)**			

***Fixed effects variables***			
Time post-exercise	0.11 (0.05)	0.01, 0.21	0.03*
Covariate: Age	0.11 (0.26)	−0.39, 0.62	0.66
Constant	45.7 (15.1)	16.1, 75.2	0.002**^†^**
***Random effects parameters***			
Intercept	82.9 (37.4)	34.3, 200.8	
Residual	15.4 (1.42)	12.8, 18.4	

***Model fit statistics***	***Statistic***		

Log likelihood	−705.7		
Bayesian information criteria	1439.0		
Pseudo *R*^2^	0.03		

**(D) Mean arterial pressure (*n* = 10, 243 observations)**			

***Fixed effects variables***			
Time post-exercise	−0.39 (0.18)	−0.75, −0.03	0.04*
Time post-exercise^2^	−0.02 (0.008)	−0.006, 0.03	0.006**^†^**
Covariate: Age	0.32 (0.25)	−0.17, 0.81	0.200
Constant	55.9 (14.5)	27.5, 84.4	<0.001**^‡^**

***Random effects parameters***	**β(*SE*)**	***95% CI***	***p***

Intercept	76.7 (35.0)	31.4, 187.4	
Residual	15.2 (1.41)	12.7, 18.2	

***Model fit statistics***	***Statistic***		

Log likelihood	−698.4		
Bayesian information criteria	1429.7		
Pseudo-*R*^2^	0.06		

*CI, Confidence interval and SE, Standard error, significant at ^∗^*p* < 0.05, **^†^***p* < 0.01, and **^‡^***p* < 0.001.*

**FIGURE 1:**
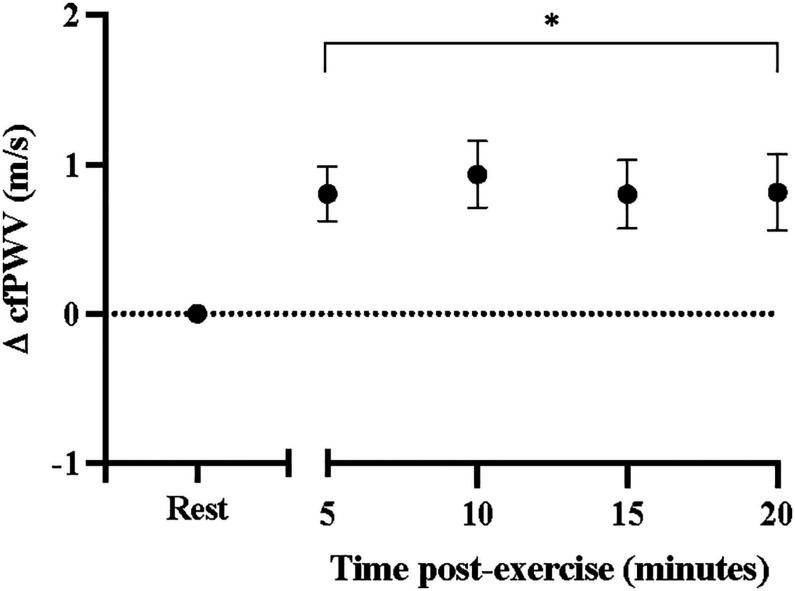
Changes in carotid-femoral pulse wave velocity at rest and after acute, high-intensity aerobic exercise. Points are presented with standard error. Significantly different from resting values *P < 0.05.

**FIGURE 2:**
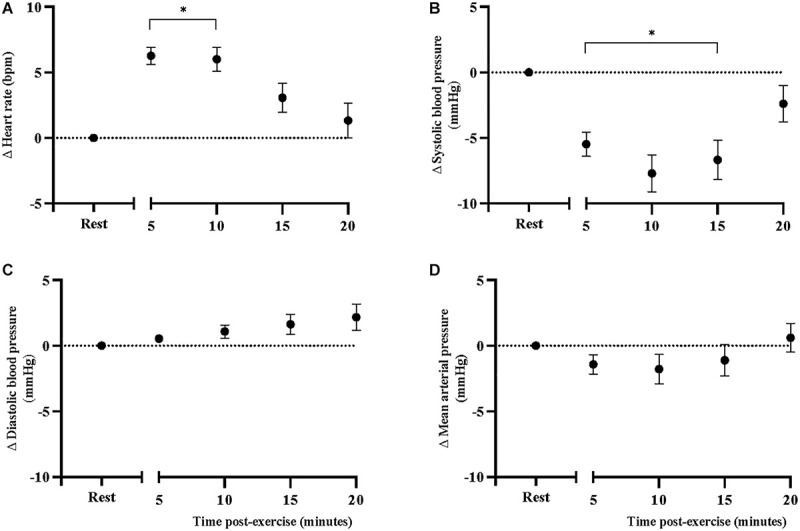
Changes in **(A)** heart rate, **(B)** systolic blood pressure, **(C)** diastolic blood pressure, and **(D)** mean arterial pressure, at rest and after acute, high-intensity aerobic exercise. Points are presented with standard error. Significantly different from resting values *P < 0.05.

Sidak-adjusted changes in cfPWV and hemodynamic variables from the mixed model analysis are presented in Table 4. Following acute aerobic exercise, cfPWV was increased from resting levels (9.0 ± 0.53 to 9.9 ± 0.52 m/s, *p* < 0.001), and remained elevated for 20 min post-exercise (Table 4), independent of changes in heart rate (*p* = 0.001, Table 2, Figure 1). Heart rate remained elevated for 10 min post-exercise (Table 4 and Figure 2A) and systolic blood pressure was reduced below resting levels for 15 min (Table 4 and Figure 2B). There were no significant differences in diastolic blood pressure and mean arterial pressure from rest (Table 4 and Figures 2C,D).

**TABLE 4:** Carotid-femoral pulse wave velocity and central hemodynamics up to 20 min after acute, high-intensity aerobic exercise.

Variable	Rest	5 min	*d*	10 min	*d*	15 min	*d*	20 min	*d*
cfPWV (m/s)	9.0 ± 0.53	9.9 ± 0.52^‡^	1.7	10.1 ± 0.53^‡^	2.1	9.9 ± 0.54^†^	1.7	9.9 ± 0.57*	1.7
HR (bpm)	71.2 ± 3.2	77.4 ± 3.1^‡^	1.9	77.2 ± 3.2^‡^	1.9	74.2 ± 3.2	0.9	72.5 ± 3.3	0.4
SBP (mmHg)	117.3 ± 4.7	111.8 ± 4.6^‡^	1.4	109.6 ± 4.6^‡^	1.6	110.6 ± 4.6^‡^	1.4	114.9 ± 4.6	0.5
DBP (mmHg)	52.1 ± 3.0	52.6 ± 2.9	0.2	53.1 ± 2.9	0.3	53.7 ± 2.9	0.5	54.2 ± 2.9	0.7
MAP (mmHg)	73.9 ± 2.9	72.5 ± 2.8	0.5	72.1 ± 2.8	0.6	72.8 ± 2.8	0.4	74.5 ± 2.8	0.2

*Values represent Mean ± SE; Significantly different from rest ^∗^*p* < 0.05, **^†^***p* < 0.01, and **^‡^***p* < 0.001. cfPWV, carotid-femoral pulse wave velocity; m/s, meters per second; HR, heart rate; MAP, mean arterial pressure; SBP, systolic blood pressure; DBP, diastolic blood pressure; and d, effect size from baseline value.*

## Discussion

Carotid-femoral pulse wave velocity remained elevated for 20 min after peak aerobic exercise in individuals with stroke. These findings were in line with our hypothesis and consistent with what has been observed in individuals with a history of chronic smoking (Doonan et al., 2011), hypertension (Gkaliagkousi et al., 2014), obesity (Bunsawat et al., 2017), and metabolic syndrome (Radhakrishnan et al., 2017). It is probable that the persisting increases in cfPWV are due to increases in sympathetic outflow to large arteries (Nardone et al., 2020). Indeed, overactivation of the sympathetic nervous system is characteristic of individuals with stroke (Dorrance and Fink, 2015), and is consistent with what has been observed in the same populations (Lambert et al., 2010; Mancia and Grassi, 2014; Middlekauff et al., 2014) that experience persistent elevations in cfPWV (Doonan et al., 2011; Gkaliagkousi et al., 2014; Radhakrishnan et al., 2017). Individuals with stroke have higher circulating levels of norepinephrine (Myers et al., 1981) and impaired heart rate recovery after exercise (Jin et al., 2013); which is indicative of autonomic dysfunction and can contribute to excessive vasoconstriction of large arteries (Nardone et al., 2020). Few studies have examined heart rate recovery in individuals with stroke, none of which have examined recovery to this length of time post-exercise. In individuals with TIA, Li et al. (2019) found that heart rate recovery was impaired at 5-min post-exercise (54 ± 16 bpm) compared to control subjects without a history of TIA (73 ± 15 bpm), at levels indicative of autonomic dysfunction. Our sample produced heart rate recovery values at 5 min post-exercise (58 ± 23 bpm) that were very similar their findings (Li et al., 2019). The sustained elevations of cfPWV and impaired heart rate recovery may be yet another indicator of autonomic imbalances in this population, especially after exercise.

Chronic high arterial stiffness increases risk of stroke (Poels et al., 2012) and is associated with poor recovery after stroke (Gąsecki et al., 2012). However, much less is known about the acute effects of elevated arterial stiffness on cardiovascular health. In young adults, cfPWV is increased immediately after exercise, but returns toward or below resting levels by 5 min (Mutter et al., 2017). In the current study, we observed immediate elevations in cfPWV of the magnitude of 1 m/s (Cohen’s *d* = 1.7) for 20 min after peak aerobic exercise. Our findings are in line with what has been observed in individuals with hypertension (Gkaliagkousi et al., 2014), except our sample sustained these elevations well into post-exercise recovery. Transient exposure to elevated cfPWV is unlikely to increase cardiovascular risk after exercise but in populations at high risk for cardiovascular disease, persisting elevations in cfPWV may be of critical importance. For instance, long-term, 1 m/s increase in cfPWV is associated with a 14–15% increase in cardiovascular events (Vlachopoulos et al., 2010). Acutely, among older adults with and without hypertension, transiently increased cfPWV is associated with cerebrovascular pulsatility (Lefferts et al., 2018; Rosenberg et al., 2020) and end-organ damage (Keith et al., 2013) following an acute bout of aerobic exercise. Prolonged exposure to elevated cfPWV may transmit pulsatile flow to the cerebrovasculature and increase the risk of cerebrovascular events in aging populations (Rosenberg et al., 2020). It is possible that individuals with pre-existing cardiovascular risk factors may be sensitive to sustained elevations in pulsatility through arterial stiffness acutely after exercise. Future studies may seek to examine the interactions between cfPWV and cerebrovascular function after acute exercise in individuals with stroke to elucidate these mechanisms.

Arguably, however, the prolonged elevations cfPWV following an acute exercise stimulus should not deter this population from engaging in exercise training. In fact, repeated exposure to exercise bouts (i.e., aerobic exercise training) creates beneficial adaptations to the autonomic nervous system (Jin et al., 2013) which may attenuate the sustained elevations in cfPWV. Indeed, 12 weeks of progressive-intensity aerobic exercise in individuals with chronic stroke was shown to improve heart rate recovery, an indicator of autonomic nervous system functioning (Jin et al., 2013). We posit that aerobic exercise training can lead to similar adaptations in the arteries, demonstrating diminished vascular responses to acute stimuli over time. Our results may be specific to this particular exercise modality (recumbent stepper). Nonetheless, our results suggest that additional caution should be considered by clinicians and researchers when community-dwelling individuals with stroke are performing peak or maximal exercise tests.

There are several strengths to this study. First, we used a symptom-limited CPET validated for individuals with stroke to examine the acute effects of exercise on cfPWV. This protocol allowed participants to reach sufficiently high intensities of exercise in spite of their physical limitations. We also utilized a comprehensive approach to the assessment of cfPWV, using the criterion-standard method of applanation tonometry (Wilkinson et al., 2010) that was also collected in a continuous manner. This method enabled the sensitivity to capture the time-dependent changes that occur during exercise recovery, in contrast to previous studies which reported arterial stiffness at discrete timepoints with time gaps of up to 10 min between measurements. This was only the second study to record post-exercise cfPWV continuously (Rakobowchuk et al., 2009), and the first study to apply these methods to a clinical population. Similarly, heart rate and blood pressure were recorded continuously and over the same time course of post-exercise recovery, thereby allowing us to concurrently examine changes in cfPWV with hemodynamic measurements. Heart rate is a major determinant of cfPWV, with cfPWV increasing 0.17 m/s for every 10 bpm increase in heart rate (Tan et al., 2016). Although we did not observe a statistically significant relationship in our cfPWV model, elevations in heart rate that can result in a distended and stiff artery due to reductions in elastic recoil time of large arteries (Mangoni et al., 1996). Therefore concurrent collection of heart rate was imperative for our analyses and allowed us to find that persisting elevations of cfPWV in individuals with stroke occurred independently of heart rate.

We acknowledge that the sample size was small, which did not allow us to adjust or disaggregate our findings by other factors such as sex and medication use in this exploratory study. It is known that there exist important sex-based differences in the cardiovascular response to exercise, as females are more susceptible to sustained elevations in cfPWV compared to males (Doonan et al., 2013). Furthermore, anti-hypertensive medication use is known to reduce the post-exercise elevations of cfPWV in individuals with hypertension (Gkaliagkousi et al., 2014). Although our small sample did not allow us to formally consider these in our models, we considered the effects of age and heart rate. Furthermore, the continuous nature of our data collection allowed us to have a higher number of observations for each variable, for each participant. Moreover, mixed model analyses are able to handle variations in time between measurement and missing data (Detry and Ma, 2016), making this an ideal approach to model continuous post-exercise changes in cfPWV. Mixed models are also preferred over repeated measures analysis of variance for small samples with repeated measurements (Muth et al., 2016). We also recognize that 20 min post-exercise may not have been long enough to observe full recovery of cfPWV in this population. However, continuous use of applanation tonometry may not be feasible beyond 20 min and may distort the quality of acquired signals. We also acknowledge that the duration of cool-down was not homogeneous across participants. However, we permitted a self-selected duration of cool down (up to 5 min) to reduce the risk of adverse effects. Furthermore, we accounted for this variability in our analyses by defining *time post-exercise* as the time between the end of the CPET and start of the hemodynamic assessment. Although it is established, in populations without stroke that cfPWV increases following exercise (Mutter et al., 2017; Pierce et al., 2018), the present study did not include a comparison group of participants without stroke, nor did we include a group that did not perform exercise, which limits the inferences that may be drawn from our findings. Future research to further examine the exercise responses of cfPWV using non-exercise and non-stroke control groups, as well as larger sample sizes, are warranted.

## Conclusion

This was the first study to model the recovery of arterial stiffness, heart rate and blood pressure following peak aerobic exercise in individuals with stroke. Aerobic exercise training is a potent strategy to reduce the risk of recurrent stroke, however, our results suggest that the elevations in arterial stiffness are persistent for at least 20 min following a single bout of peak-intensity exercise, independent of heart rate. These preliminary findings may provide evidence of excessive sympathetic activity in individuals with stroke.

## Data Availability Statement

The raw data supporting the conclusions of this article will be made available by the authors, without undue reservation.

## Ethics Statement

The studies involving human participants were reviewed and approved by Hamilton Integrated Research Ethics Board (HiREB), McMaster University. The patients/participants provided their written informed consent to participate in this study.

## Author Contributions

KN and AT conceived the study. KN contributed to data collection, analysis and interpretation, and led the manuscript preparation. KM and EW contributed to data collection, analysis, and the manuscript preparation. JR and MM contributed to the manuscript preparation. MR and AT secured research funding for this study and contributed to the manuscript preparation. All authors contributed to the article and approved the submitted version.

## Conflict of Interest

The authors declare that the research was conducted in the absence of any commercial or financial relationships that could be construed as a potential conflict of interest.
